# Mutual Interaction between Plasma Characteristics and Liquid Properties in AC-driven Pin-to-Liquid Discharge

**DOI:** 10.1038/s41598-018-30540-4

**Published:** 2018-08-13

**Authors:** Sung-Young Yoon, Hyeongwon Jeon, Changho Yi, Seungil Park, Seungmin Ryu, Seong Bong Kim

**Affiliations:** Plasma Technology Research Center of National Fusion Research Institute, 37, Dongjangsan-ro, Gunsan-si, Jeollabuk-do 54004 South Korea

## Abstract

This study investigated the mutual interaction between the plasma and plasma treated water (PTW). Many works have shown that the plasma treatment decreases the pH of PTW due to nitric oxide electrolyte ion but the interactions between PTW and the plasma are still largely unknown. We investigated the effect of PTW on a plasma as well as the effect of a plasma on PTW using a pin-to-liquid discharge system. It is found that PTW affects not only the chemical properties but also the physical properties of the plasma such as breakdown voltage and concentration of plasma column. The decrease of the liquid surface tension of PTW due to nitric oxide electrolyte ion from the plasma results in the increase of plasma current onto the surface of PTW and vice versa. The feedback process will be continued until the transition from normal discharge to abnormal discharge. These results can be basic data for the development of plasma sources to treat liquids.

## Introduction

In recent decades, plasma application has broadened from the semiconductor industry to living organisms: surgery^1^, meat treatment^2^, microorganism removal in sea water^3^, plasma-based water purification^4^, periodontitis treatment^5^, and agriculture^6^. In particular, pin-to-liquid discharge generates abundant nitric oxide species from humid air. This is well-known as nitrogen fixation, which is important for fertilization in agriculture. In biological and agricultural applications, the interactions between plasmas and surfaces of liquid targets are very important issues. It is well-known phenomena that impurities from the treatment target change the characteristics of low-pressure plasmas like electron density and electron temperature^7^. Furthermore, the effects on plasma characteristics are more severe in the case of a liquid target rather than a solid target because of density change of electrolyte ions in the liquid target, easy evaporation from the liquid target surface and the surface deformation of the liquid target.

In our previous work^8–11^, we found the liquid target is also an important factor to determine the characteristics of plasmas. We have focused on the transition of plasma and liquid properties during the long-term over tens of minutes^10^. The role of PTW in electronic components is as a resistor, so the solid electrode-to-liquid electrode discharge system is called as a “resistive barrier discharge”^12^. Plasma dissolves electrolyte ions into the plasma treated water (PTW). The dissolved electrolyte ions increase the electrical conductivity of PTW^10^. The increase of PTW electrical conductivity means a low-current resistor; thus, the plasma can transit from glow to arc via abnormal. The long-term transition of plasma and liquid properties is important in both the understanding of the plasma discharge mechanism and the development of plasma sources for applications. We also studied the transition of the plasma shape from a ring-shaped bullet to a pin-like streamer adjacent to a liquid surface due to the evaporation of the liquid^11^. The difference of ionization energy or the attachment rate of evaporated H_2_O to ambient discharge gas species is also able to change the plasma. This transition can be observed adjacent to the liquid surface. When the PTW temperature increases by Joule heating from the discharge current, the enhanced evaporation expands the transition region.

A number of works have reported the distortion of the liquid surface by plasmas. Sommers *et al*. generated a streamer discharge in a bubble and observed the oscillation of the bubble surface^13^. They reported that the oscillation of the bubble surface was driven by the electric field induced by the streamer from the analysis of Kelvin’s equation. Regarding the effects of the electric field on the liquid surface, Bruggeman *et al*. reported that an electric field from an electrode caused to the deformation of the water surface^14^. The deformed profile was measured by using a cathetometer, and it agreed well with the calculated profile from the force balance equation. However, the force balance should include not only electric force but also surface tension of the liquid. The surface profile of PTW will be differently deformed even under constant electric field as the electrolyte ion density in PTW is varied, because a dissolved electrolyte ion is able to change the surface tension of PTW. The surface deformation will result in the decrease or the increase of the gap distance between the electrode and the liquid surface. The change of the gap distance is an important issue from the point of view of plasma source development. For example, the gap distance is important to decide the breakdown voltage. In atmospheric pressure discharge at the low driving frequency, plasma on and plasma off repeat every a cycle. If the change of the gap distance is less than a millimeter during plasma treatment, the breakdown voltage can be varied in hundreds of volts. Thus, one should be able to quantitatively anticipate the gap distance in a given treatment target, especially liquid target.

In this work, we evaluate the surface tension of PTW with the electrical conductivity of PTW and investigate the effect of surface tension on plasma properties. We prepare PTW samples with different conductivity by changing the treatment time. We focus on the relation between plasma properties and the electrical conductivity of PTW, not the treatment time because the electrical conductivity is a more general parameter than the treatment time to present the property of PTW. The results are presented in the next section and in Section III, we briefly conclude the mutual interaction between the plasma characteristics and the properties of PTW. The detailed setup and the numerical calculation of the deformed liquid surface are described in the method section.

## Results

### Properties of PTW

**Figure 1:**
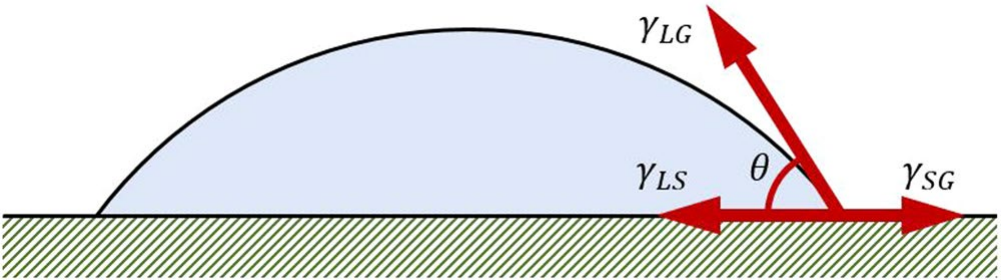
Contact angle and surface tensions between solid, liquid, and gas as related by Young’s equation.

Four samples of the PTW, which were prepared as the treatment time was varied from 0 to 20 minutes in the pin-to-water discharge, have different properties with pH and electrical conductivity as shown in Table 1. The longer plasma treatment causes the lower pH and high electrical conductivity in water. It is noted that we used the multi pins-to-water discharge system not a single pin-to-water discharge system for the sake of time-saving. In the method section, the preparation process of the PTW samples is described in more detail. The contact angles of the PTWs are decreased with plasma treatment time on all surfaces of the three materials: acryl, glass, and alumina. The force balance of surface tensions (Fig. 1) for the contact angle *θ* can be expressed as Young’s Equation (1)

1 $${\gamma }_{SG}={\gamma }_{LS}+{\gamma }_{LG}\,\cos \,\theta $$

or

2 $$\cos \,\theta =\frac{({\gamma }_{SG}-{\gamma }_{LS})}{{\gamma }_{LG}}$$

**Table 1:** Properties of PTW with pre-treatment time.

		Case 1	Case 2	Case 3	Case 4
Pre-treatment time (min.)		0	5	10	20
Electrical conductivity (μS/cm)		0.07	11.6	55.1	440
pH		7.0	4.75	4.02	3.01
Contact angle (°)	Acryl	77.20	70.37	66.31	61.53
	Glass	70.94	65.31	61.50	59.14
	Alumina	64.74	52.58	50.94	47.07

where γ_SL_, γ_LG_, and γ_SG_, are the solid-liquid interfacial surface tension, liquid-gas surface tension, and solid-gas free energy, respectively. The surface tension of liquid γ in Equation (5) is γ_SL_. The contact angle *θ* decreases with *γ*_SL_^15^. As shown in Fig. 2, the contact angle decreased with longer treatment time (i.e., higher electrical conductivity) in all three materials. Thus the decrease of contact angle in Table 1 and Fig. 2 imply the decrease in surface tension. The variation of surface tension differs between the electrolyte species^16^. It implies that the trends of surface tensions with plasma treatment time could be different depending on discharge gas species. In the pin-to-liquid system, the major dissolving electrolyte from the air plasma is HNO_3_^10^, which decreases the surface tension. If the discharge system dissolves ions such as LiCl, KOH, NaCl, NaBr, CsCl, NH_4_Cl NH_4_NO_3_, or NaI into the PTW, surface tension will increase^16^.

**Figure 2:**
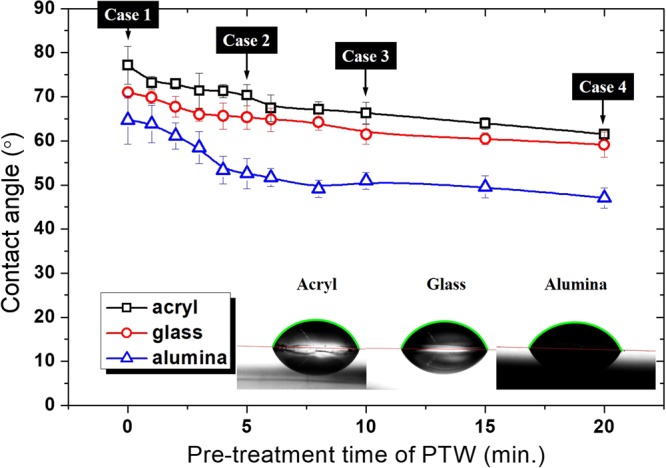
Contact angles of plasma treated water on the surface of acryl, glass, and alumina.

### Deformation of PTW surface

**Figure 3:**
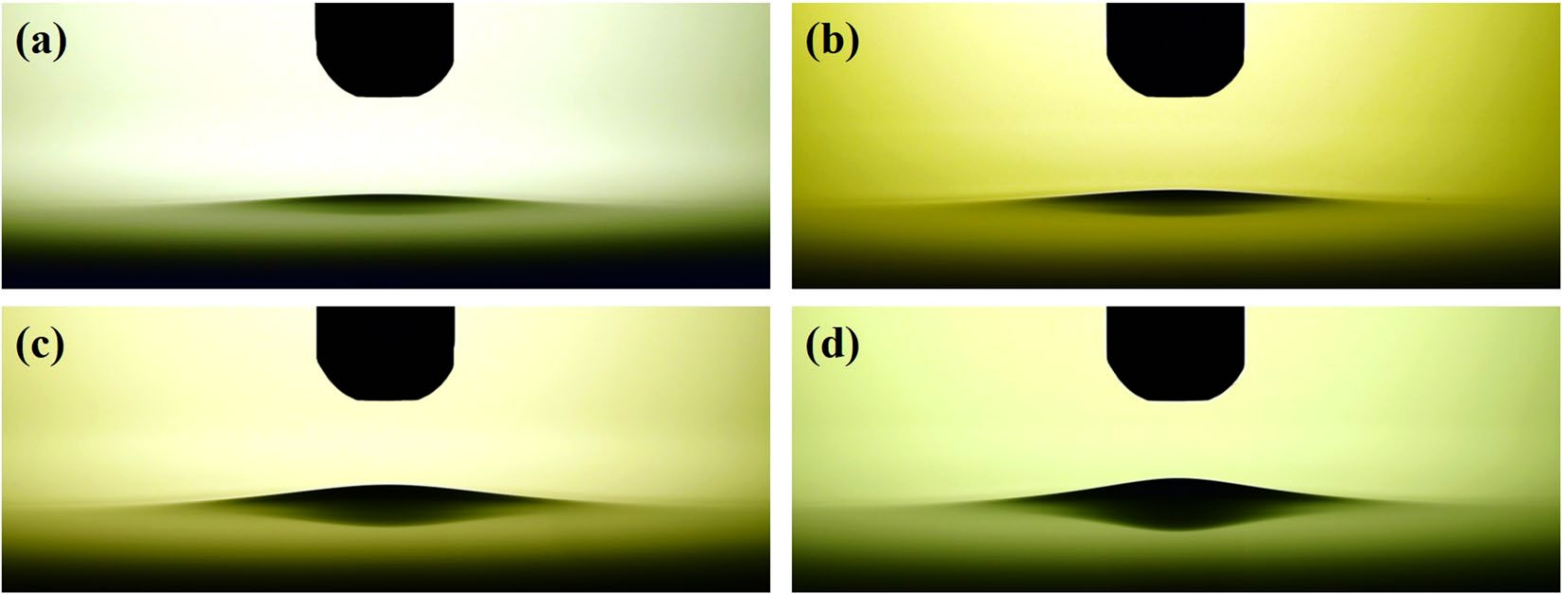
Images of deformed water surface by the electric field. The applied voltage condition is fixed in 12.5 kHz ac-driven 3 kV_pp_. The PTWs in each image is (**a**) case 1, (**b**) case 2, (**c**) case 3 and (**d**) case 4.

**Figure 4:**
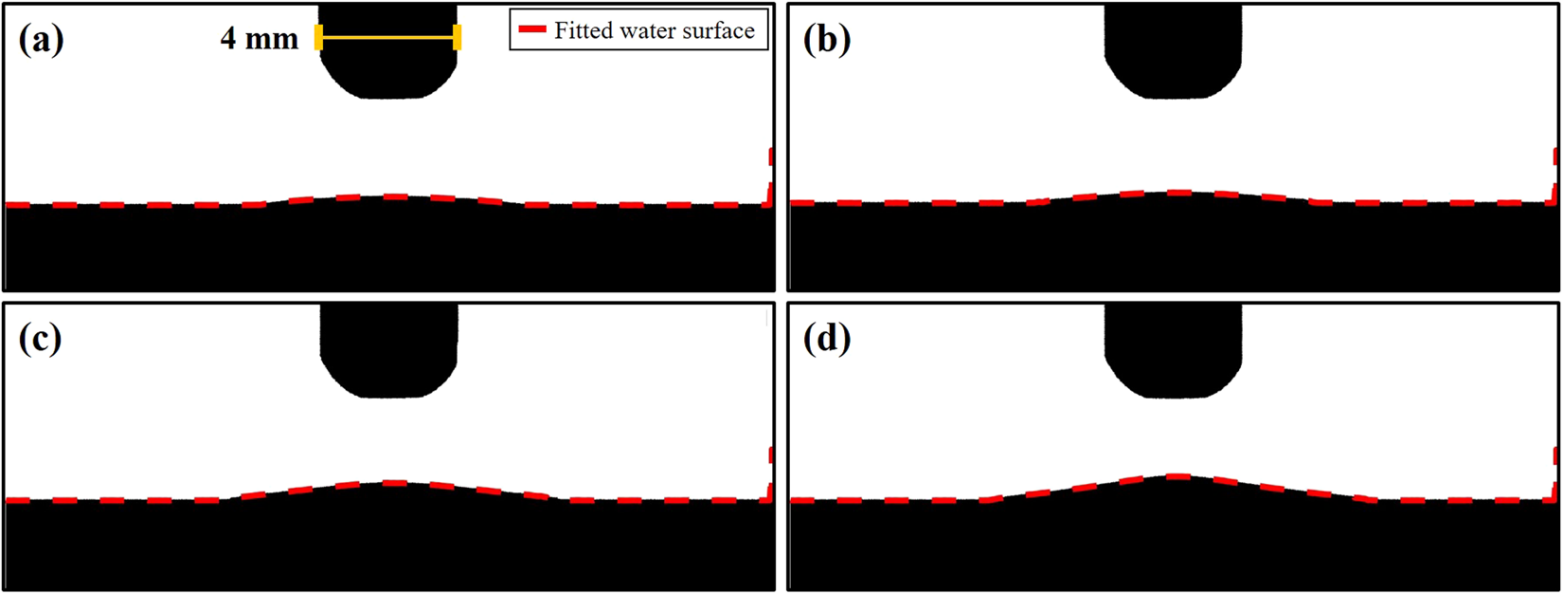
High contrast and black and white images from Fig. 3 to evaluate the profiles of deformed water surface. The dashed red lines indicate the evaluated liquid profiles. The reference distance, 4 mm, in the images is obtained from the diameter of the electrode.

**Figure 5:**
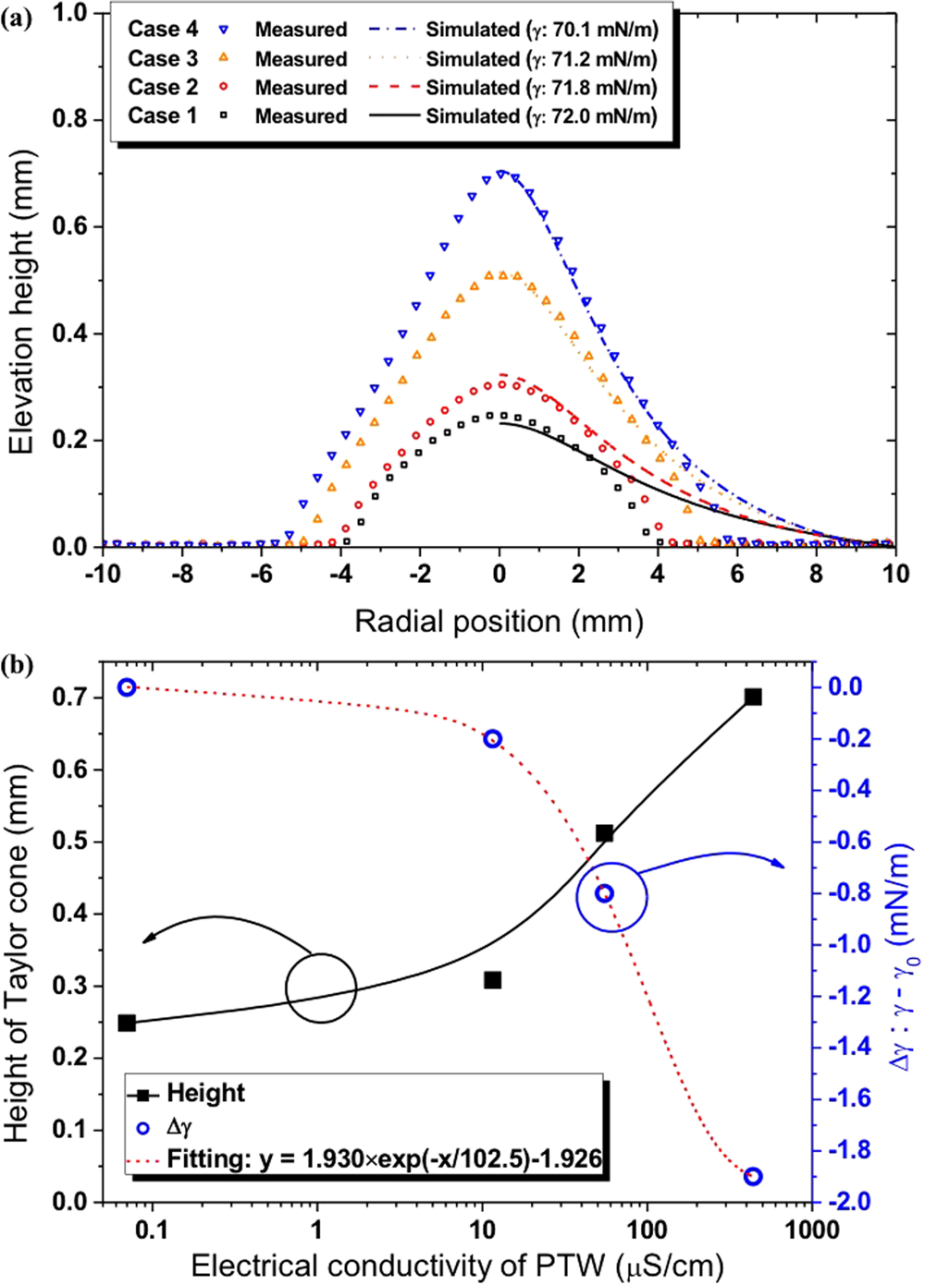
(**a**) Measured and simulated deformed water surface profile and (**b**) height of Taylor cone and surface tension change with the electrical conductivity of PTW.

Figure 3 shows the images of the surface deformation of the prepared PTWs under the conditions of driving frequency of 12.5 kHz and a peak-to-peak voltage of 3 kV_pp_. The plateau time of the applied square-shape voltage was 0.4 ms as shown in Fig. 3 and the exposure time for the images was 10 ms. Suvorov *et al*. reported that it takes less than 1 μs to saturate the surface deformation of a liquid under the constant voltage and the surface deformation is relevant to the amplitude of electric field not to voltage polarity^17^. Thus, it is possible to assume Fig. 3 represents the saturated surface deformation of the PTWs. Figure 4 shows the surface profiles of the PTWs deduced from the images in Fig. 3. We decreased the colorfulness and increased the contrast of the images to make a clear distinction between the liquid and the background. The dashed red lines indicate the evaluated profile. The reference distance, 4 mm, was obtained from the diameter of the electrode, as indicated in Fig. 4(a). The errors in evaluation at the right end of the images were cropped. In the method section, model and simulation of the surface profile of the deformed PTWs are described in detail. In the Equation (5), the electric field is estimated from the given voltage on the electrode. The surface tension of the liquids is obtained by fitting the surface tension γ from the Equation (5) to the measured liquid surface profile. The measured and simulated results are presented in Fig. 5(a). The simulated results show good agreement with the measured results. The disagreement near the liquid surface might be caused during the image processing of the contrast and colorfulness. This method may be able to measure the surface profile when the surface deformed more than 0.1 mm. It is clear that the PTW shows low surface tension compared to the untreated deionized water. The surface tension and heights of the Taylor cone with the electrical conductivities are presented in Fig. 5(b). As the electrical conductivity of PTW increased from 0.07 to 440 μS/cm, the surface tension of PTW exponentially decreased from 72.0 to 70.1 mN/m.

### Plasma characteristics with PTW

**Figure 6:**
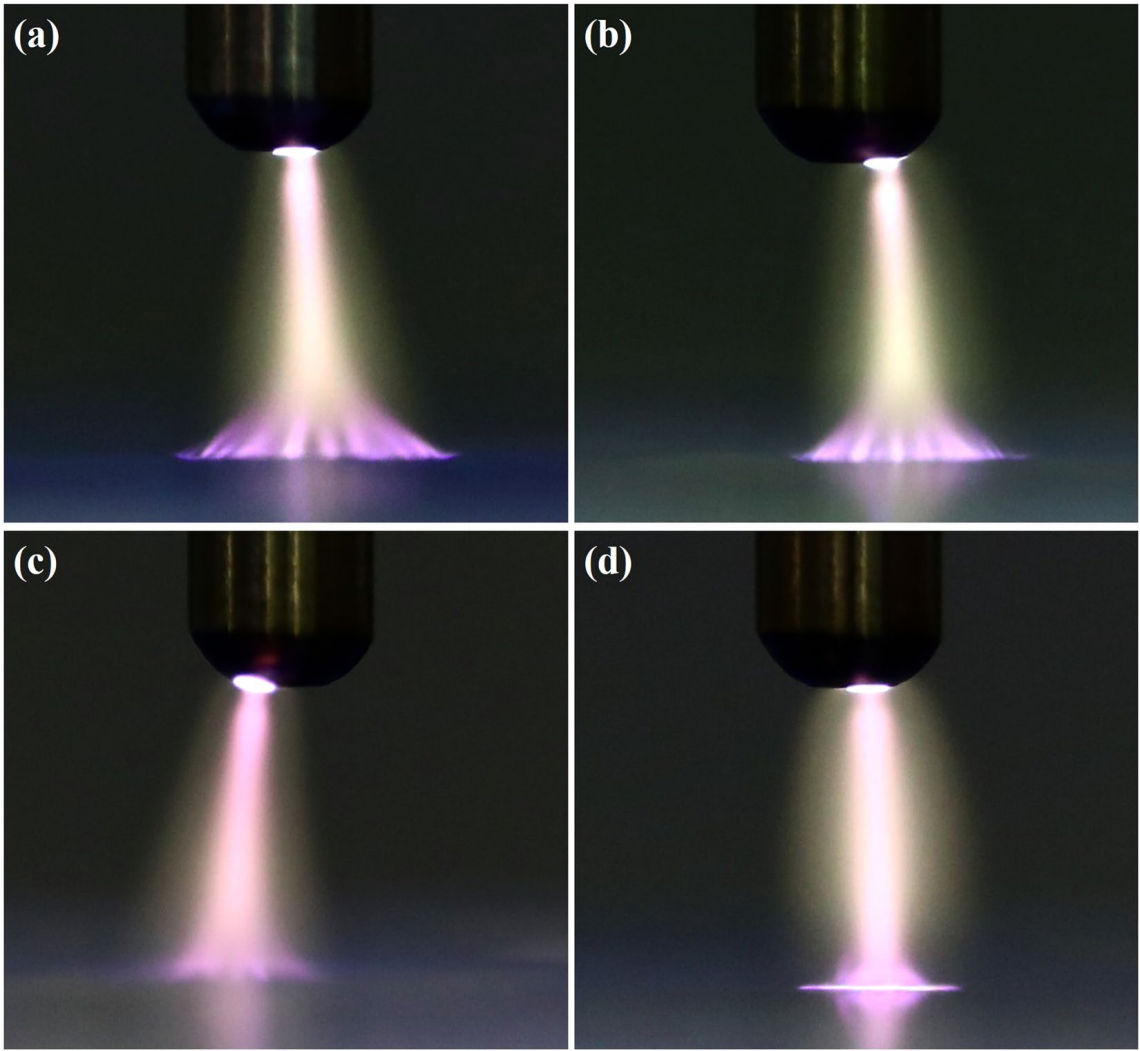
Images of the plasma for conditions of PTW: (**a**) DI water (case 1), (**b**) case 2, (**c**) case 3, and (**d**) case 4. The applied voltage condition is fixed in 12.5 kHz ac-driven 4 kV_pp_.

**Figure 7:**
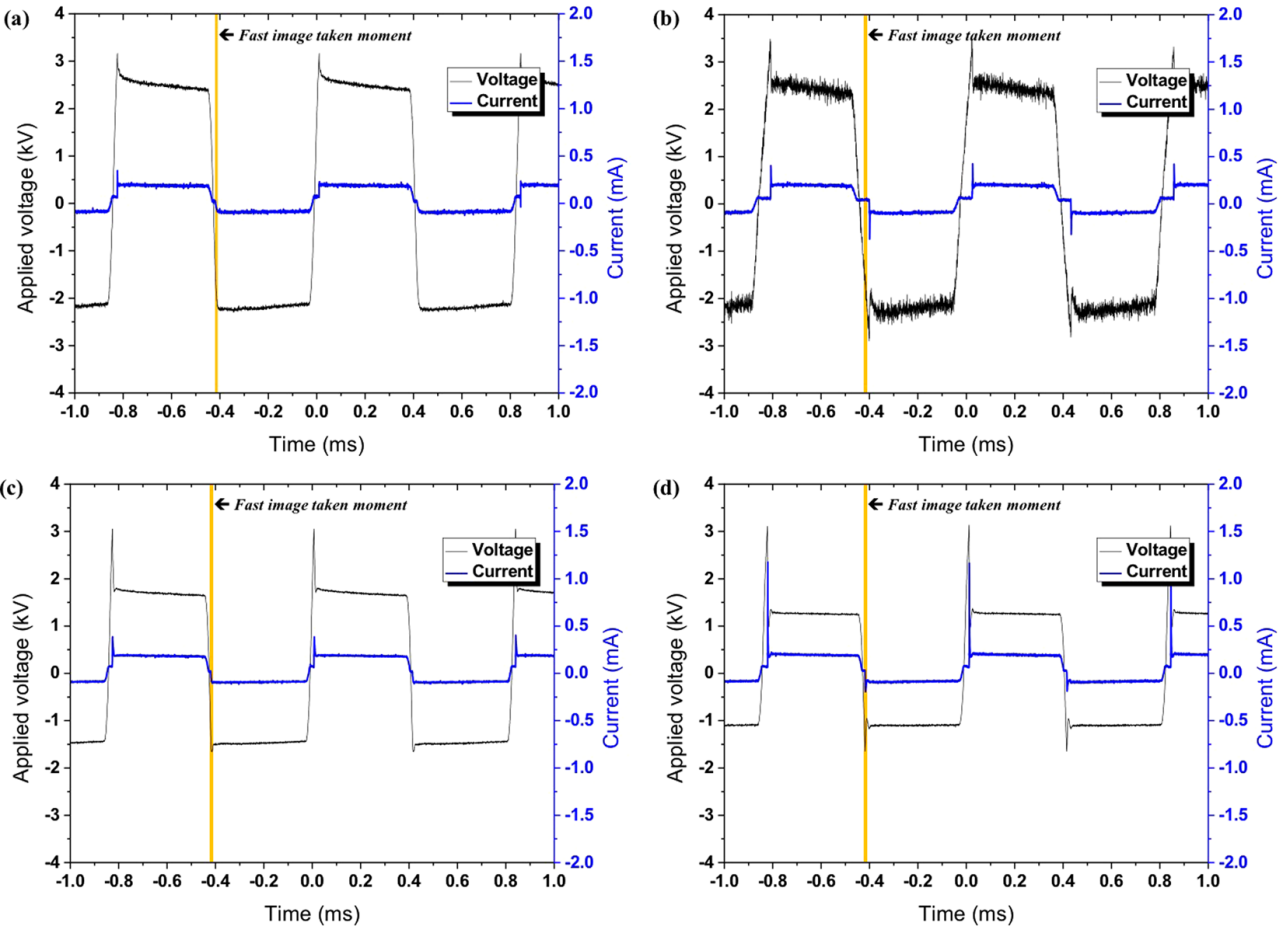
V-I signals for conditions of water: (**a**) DI water (case 1), (**b**) case 2, (**c**) case 3, and (**d**) case 4.

**Figure 8:**
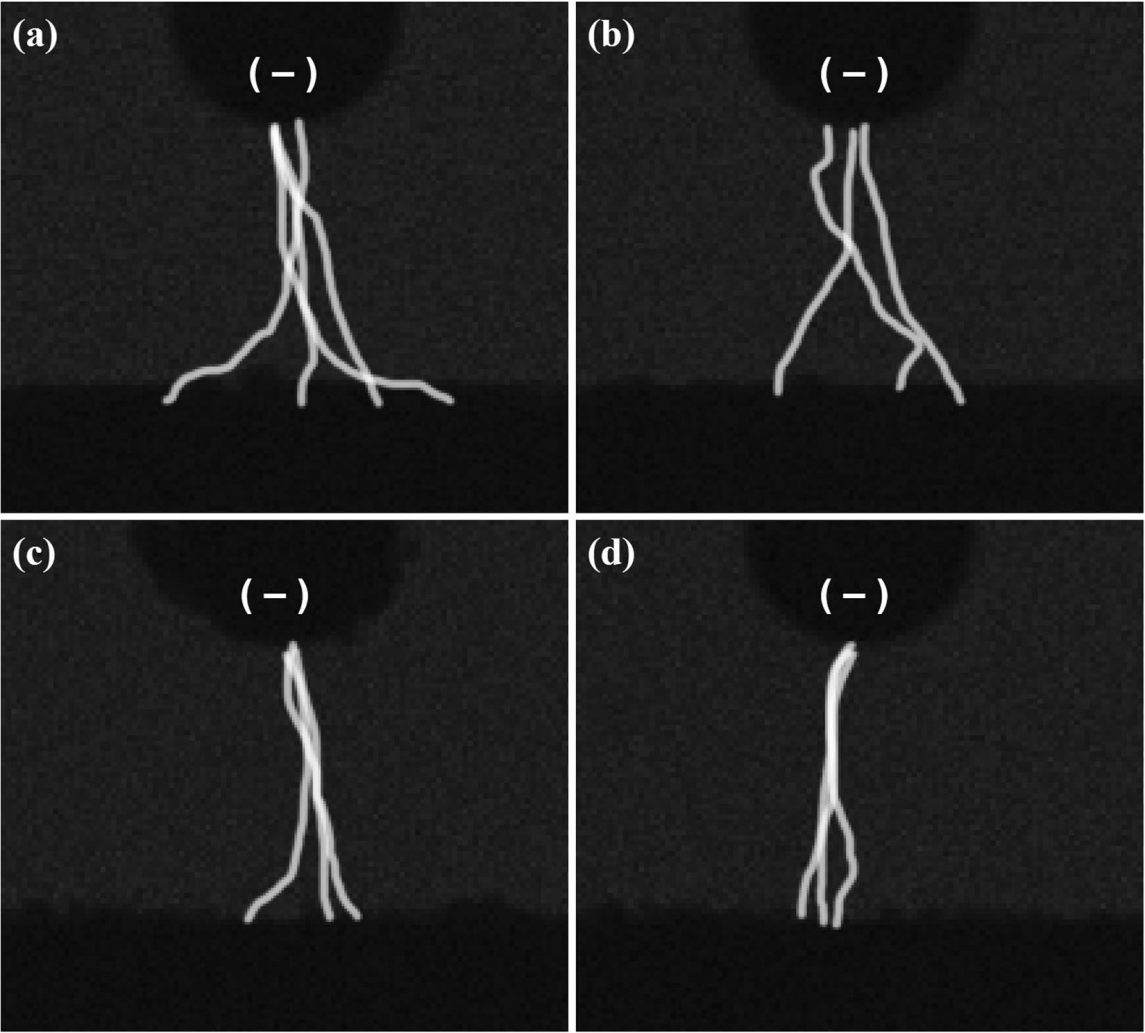
The merged high-speed images of three plasma columns for conditions of water: (**a**) DI water (case 1), (**b**) case 2, (**c**) case 3, and (**d**) case 4.

**Figure 9:**
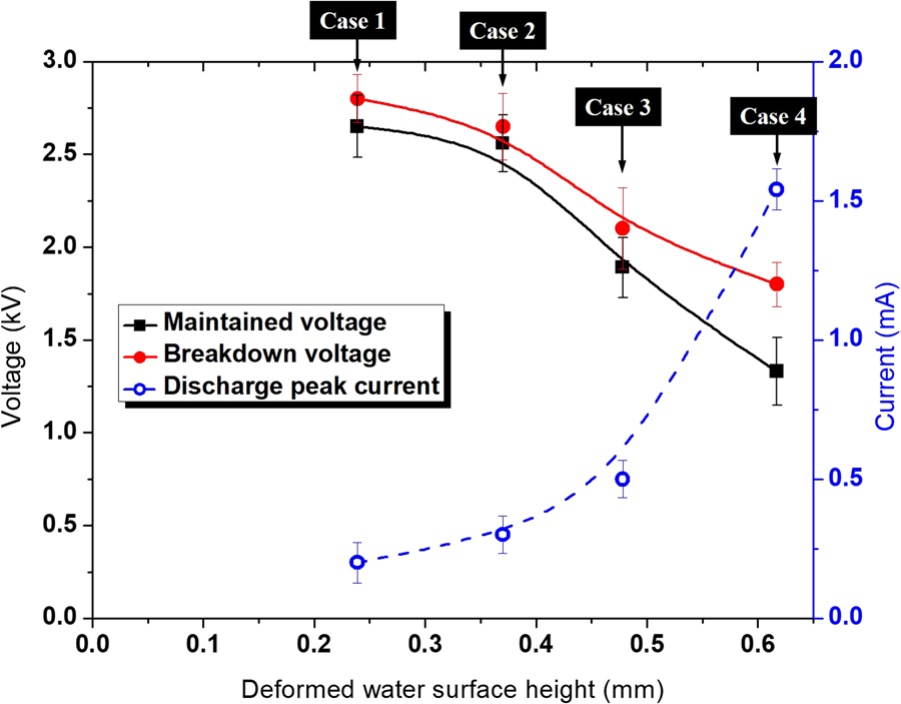
Maintained voltage, breakdown voltage, and discharge peak current with deformed water surface height.

Plasmas were generated between the electrode and the PTW surfaces with the different electrical conductivities as shown in Fig. 6(a–d) when the applied voltage was 4 kV_pp_ at the frequency of 12.5 kHz. It is noted that no plasma was generated between the electrode and the PTW surfaces under the condition of driving frequency of 12.5 kHz and a peak-to-peak voltage of 3 kV_pp_ as shown in Fig. 3(a–d). The plasmas have the typical structure of the anode (or cathode) spot – positive column, surface pattern on a liquid surface. This branching of the plasma column on the PTW surface and the concentration of pattern area with electrical conductivity are consistent with the reported work by Verreycken *et al*.^18^. They reported that the high electrical conductivity decreases the charge relaxation time in a liquid and the patterned anode spot implies the accumulation of plasma charge on the liquid surface. Figure 7 shows the V-I signals of plasma discharge according to PTW conditions. Figure 8 shows the fast images of the discharges at the moment marked in Fig. 7. The fast images were taken when the applied voltage polarity is negative. It is noted that the polarity in Fig. 6 cannot be determined because the exposure time of the images is longer than the period of applied ac-driven voltage. In the case of the deionized water, in case 1, the individual plasma columns spread on the deionized water surface and the column is bent rather than branching. On the other hand, the plasma columns become straighter as the conductivity of PTW is higher. It supports the hypothesis that the anode pattern is formed due to the accumulated charge on the liquid surface. As the charge accumulation decreases, the plasma column is concentrated and straightened. Figure 9 shows maintained voltage, breakdown voltage and discharge peak current obtained from Fig. 7. As the conductivity of the PTWs becomes higher, the discharge peak current increases and the discharge peak current causes the maintained plateau voltage to be lower. Thus, the PTWs with different electrical conductivity results in different structures and concentrations of plasma columns and affect the discharge characteristics such as maintained voltage, breakdown voltage, and discharge peak current.

### Mutual interaction between Plasma and PTW

**Figure 10:**
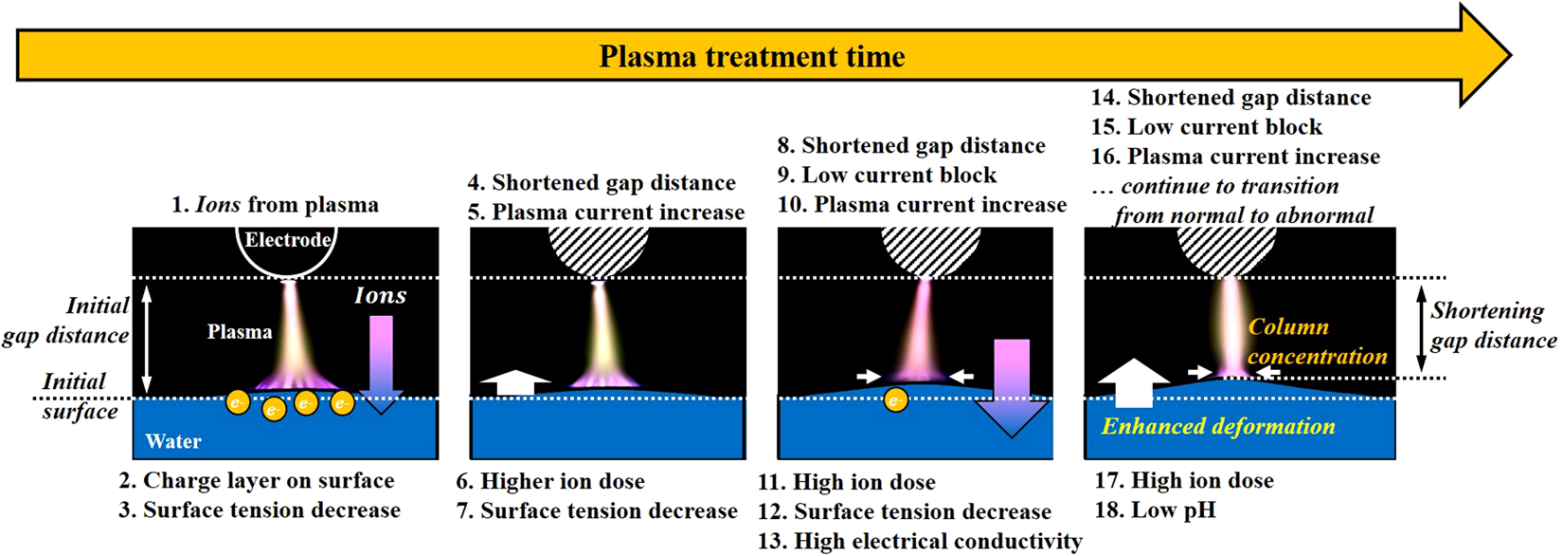
Mutual interactions between the plasma and water.

If we keep plasma treatment on the PTW in the case 4 for a long time, what will happen? The concentration of plasma column causes the discharge current to be higher and the conductivity of the PTW becomes higher. The PTW with higher conductivity increases again the discharge current. The feedback process will cause the plasma to transit from glow to arc via abnormal. In our previous work, we reported the time-delayed plasma transition from glow to arc^10^. Based on the results, therefore, we suggest the concept of the mutual interactions between the plasma and water as shown in Fig. 10. The plasma discharge causes the electrolyte ion to dissolve in the PTW. The atmospheric pressure plasma discharge system is sensitive in the discharge gap distance. In the case of the plasma treatment generating PTW from deionized water, for example, the enhanced Taylor cone height will significantly change the plasma properties. As shown in Fig. 9, the breakdown voltage decreased by 33% when the pH of PTW reached ~3.0 during plasma treatment. Note that any external control factors not changed. The decreased gap distance caused the lower breakdown voltage and the higher discharge current. The enhanced deformation increased the plasma current. The higher plasma current leads to the higher electrolyte ion dissolving. Thus, the plasma discharge current gradually increased with the mutual interaction between the plasma and the facing liquid.

## Conclusion

In this work, we investigated the mutual interactions between the plasma characteristics and PTW properties in pin-to-liquid discharge system. The electrolyte HNO_3_ from the plasma discharge decrease the surface tension of water. The deformation of the liquid surface increase with the lower surface tension of the liquid. The HNO_3_ transits to H^+^ and NO_3_^−^ in the PTW, and increases the electrical conductivity of PTW, causing the higher discharge current. These two changes (increase in electrical conductivity and decrease of surface tension) in liquid properties synergistically increase the discharge current and enhance the surface deformation and vice versa. Since the atmospheric pressure discharge is sensitive to the gap distance, the shortened gap distance due to the surface deformation even causes the plasma discharge transition: from glow-like discharge to spark-like discharge. Thus, the properties of plasma and liquid are decided not by independent but with mutual interactions. It is expected that the results of surface tension decrease with the electrical conductivity of PTW can be important basic data to decide the gap distance in discharge system for liquid treatment such as the fluid dynamic analysis in lab-on-a-chip devices.

## Methods

### PTW preparation

The PTWs with different pH and electrical conductivity were prepared by treatment time in the pin-to-water discharge. The 1.5 L of purified water is treated by plasma. After 5, 10 and 20 min. of treatment, the 1 L of PTW is used to measure the surface tension of the PTW and plasma characteristics on the PTW. The non-treated water is referred to as case 1 in this work. Case 2–4 represent the 5–20 min. treated PTW. The detailed dimension and plasma generating power are described in our previous work^10^.

### Plasma Generation and monitoring

**Figure 11:**
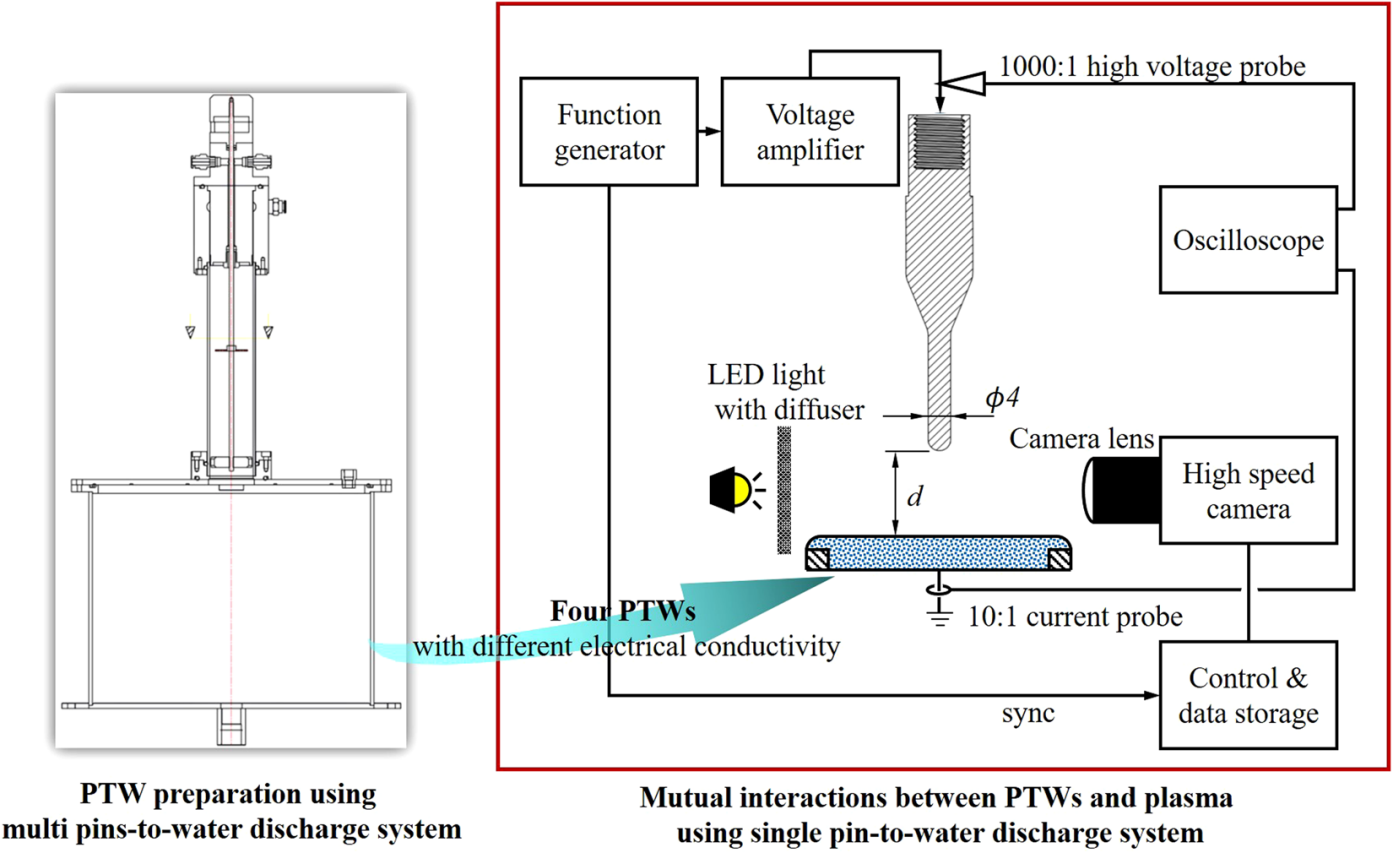
Diagram of the experimental setup. The *d* indicates the initial gap distance between the metal pin electrode and un-deformed water surface.

The experimental setup is shown in Fig. 11. A 4-mm diameter electrode with a spherical end of 2 mm curvature electrode was placed ~3 mm above 1000 mL of PTW in a reservoir. In this work, we distinguish the PTW from the untreated deionized water. Thus, we use the term “liquid” as the deionized water and the PTW. The electrical conductivity of the PTW was controlled by treatment time. A longer treatment caused lower pH and higher electrical conductivity^10^. The pH and electrical conductivity of the untreated deionized water were 0.07 μS/cm and 7.0, respectively. The range of electrical conductivities of the different PTW samples was 11.5–440 μS/cm with corresponding pH levels of 3.01–4.75. The distance between the electrode end and the PTW surface is noted as *d* in the figure. The *d* was carefully adjusted with a micrometre to perform the experiment under a constant voltage of 3.0 kV in the PTW surface deformation measurement. The voltage was increased to 4.0 kV in plasma generation. The surface of the PTW rises in a Taylor cone shape when we applied a high voltage with a voltage amplifier (Trek, 20/20 C) with a function generator (Tektronix, AFG3000) on the metal electrode. When *d* was too small, the deformed PTW contacted with the powered electrode. If *d* was too large, the plasma was not discharged at the maximum voltage from the voltage amplifier.

We estimated the surface tension of PTW by monitoring the profile of the liquid using a digital camera (Canon, EOS-1D X Mark II) with a macro lens (Canon, EF 180 mm f/3.5 L Macro USM) and background light (Thorlabs, M590L3 and LEDD1B). The image taken condition is f-number 3.5, iso of 5000 and exposure time of 1/500 s, respectively. The surface tensions of the PTW were obtained by comparing the measured deformed surface profiles to the simulated ones. The details of the simulation are described in the next sub-section. The corresponding characteristics of the plasma were observed from the discharge images and V-I signals. The V-I signals were monitored using a high-voltage probe (Tektronix, P6015A) and a current probe (Pearson, model 411). Discharge images with a short exposure time were obtained from an ultra-high-speed camera (NAC image technology, ULTRANAC Neo) with an F-mount imaging lens (Nikon, AF-S VR Micro Nikkor ED 105 mm f/2.8 G (IF)). The moment of exposure was controlled from the sync signal of the function generator. In each measurement, three images were obtained with an exposure time of 0.5 μs. After taking the fast images at same voltage phase, we merged the images by ‘lighter color’ in Photoshop blending mode.

The discharged plasma is able to change the PTW ion concentration during the plasma characteristics measurement. To minimize the effect of plasma on the PTW during the measurement, each measurement was carried out within 5 min. The change in electrical conductivity of the PTW was less than 5% compared to the values of the PTW before the measurement.

### Modeling of the water surface deformation

**Figure 12:**
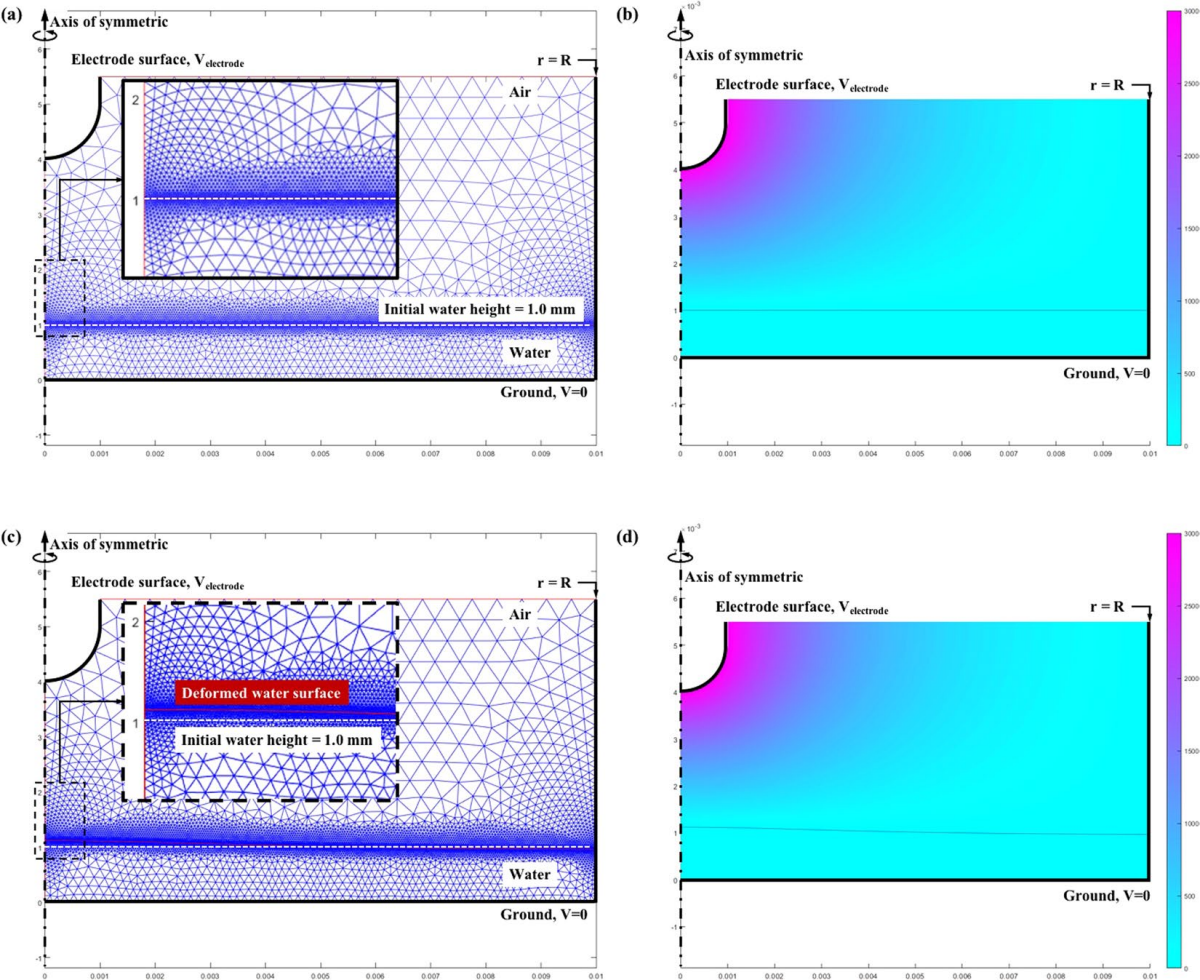
(**a**) The initial geometry, computational mesh, and boundary conditions in the calculation (**b**) initial distribution of electrical potential, (**c**) updated geometry and computational mesh at the end of the calculation, and (**d**) electrical potential distribution in the updated geometry.

As mentioned before, we estimated the surface tension of the PTW by comparing the measured deformed PTW surface profile to the simulated profiles. We calculated the deformed PTW surface from the Laplace equation and force balance equation. The domain and mesh for the numerical calculation are presented in Fig. 12(a). The liquid was considered as an electrically neutral material with finite permittivity. The electrical potential distribution is obtained in air and liquid from the Laplace equations:

3 $$\nabla \cdot (-{{\epsilon }}_{0}\nabla {\rm{\Phi }})=0\,{\rm{for}}\,{\rm{air}}\,{\rm{and}}$$

4 $$\nabla \cdot (-{{\epsilon }}_{0}{{\epsilon }}_{r\_w}\nabla {\rm{\Phi }})=0\,{\rm{for}}\,{\rm{the}}\,{\rm{liquid}}$$

where ${{\epsilon }}_{0}$, ${{\epsilon }}_{r\_w}$, and Φ(*r*, *z*) are permittivity of air 8.854 × 10^−12^ F/m and relative permittivity of water 80, and electrical potential, respectively. Figure 12(b) shows Φ(*r*, *z*) in the case of the initial gap distance and applied voltage of 3 kV. The deformed liquid surface profile is estimated from the force balance equation in the normal direction to the liquid surface,

5 $$\rho gh(r)=\frac{1}{2}{{\epsilon }}_{0}{E}_{n}^{2}(r)-\gamma \frac{|h^{\prime\prime} (r)|}{{[1+{(h^{\prime} (r))}^{2}]}^{\frac{3}{2}}},$$

where *ρ*, *g*, *h*(*r*), ${E}_{n}^{2}$, and *γ* are the density of liquid 1 kg/m^3^, gravity acceleration 9.8 m/s^2^, liquid surface height profile, square of normal electric field at the liquid surface, and surface tension, respectively. *E*_n_ is obtained from Equations (3) and (4). The surface tension of pure water is 71.97 mN/m at room temperature. Equation (5) is numerically solved by using MATLAB^®^ (ver. 2017b). The boundary conditions of Equation (5) are taken as

6 $${\frac{d}{dr}h(r)|}_{r=0}=0$$

and

7 $${\frac{d}{dr}h(r)|}_{r=R}=0$$

where the R is the simulation domain radius, 1 cm in this work. The deformation of the liquid surface means the computational domain for the interface of the air and the liquid should be changed during the calculation. The updated domain and mesh are presented in Fig. 12(c). Then, Φ(*r*, *z*) is calculated with the deformed liquid surface as shown in Fig. 12(d). We repeated the calculation until saturation of the surface profile. The surface profile is considered saturated when the averaged sum of differences between the liquid surface from the current step and the previous step is less than the threshold value Δ_*thr*_, 1e-5 in this work:

8 $$\frac{{\sum }_{j=1}^{N}[h{({r}_{j})}^{(i)-th}-h{({r}_{j})}^{(i-1)-th}]}{N} < {{\rm{\Delta }}}_{thr},$$

where *N* (=400 in this work) is the node number of the liquid surface domain.
